# Autophagy Modulation and Its Implications on Glioblastoma Treatment

**DOI:** 10.3390/cimb45110546

**Published:** 2023-10-29

**Authors:** Johnny Chen, Andrea Salinas Rodriguez, Maximiliano Arath Morales, Xiaoqian Fang

**Affiliations:** 1 Department of Neuroscience, School of Medicine, University of Texas Rio Grande Valley, Edinburg, TX 78539, USA; johnny.chen01@utrgv.edu; 2 Department of Health and Biomedical Sciences, University of Texas Rio Grande Valley, Edinburg, TX 78539, USA; andrea.salinasrodrigue01@utrgv.edu; 3 Department of Biology, College of Science, University of Texas Rio Grande Valley, Edinburg, TX 78539, USA; maximiliano.morales01@utrgv.edu

**Keywords:** autophagy, glioblastoma, treatment, JAK2/STAT3 signaling pathway, PI3K/AKT/mTOR signaling pathway

## Abstract

Autophagy is a vital cellular process that functions to degrade and recycle damaged organelles into basic metabolites. This allows a cell to adapt to a diverse range of challenging conditions. Autophagy assists in maintaining homeostasis, and it is tightly regulated by the cell. The disruption of autophagy has been associated with many diseases, such as neurodegenerative disorders and cancer. This review will center its discussion on providing an in-depth analysis of the current molecular understanding of autophagy and its relevance to brain tumors. We will delve into the current literature regarding the role of autophagy in glioma pathogenesis by exploring the major pathways of JAK2/STAT3 and PI3K/AKT/mTOR and summarizing the current therapeutic interventions and strategies for glioma treatment. These treatments will be evaluated on their potential for autophagy induction and the challenges associated with their utilization. By understanding the mechanism of autophagy, clinical applications for future therapeutics in treating gliomas can be better targeted.

## 1. Introduction

Gliomas are considered the most common primary brain tumors in adults, and over half of all brain tumors are gliomas [1,2,3,4]. Glioblastoma multiforme (GBM) is regarded as one of the most aggressive and lethal types of gliomas [5,6,7,8]. Unfortunately, the cause of the disease is unknown in most GBM patients [9]. The current standard of care treatment includes surgical resection followed by radiotherapy and chemotherapy. Even after treatment, patients with GBM typically have a median survival of approximately 14 months [10,11,12]. In recent studies, glioblastoma has been classified into three subtypes based on transcriptome analysis of the GBM tumors: proneural, classical, and mesenchymal [13,14]. The mesenchymal subtype of GBM is associated with the worst prognosis due to its expression of neural stem cell markers and the creation of an inflammatory microenvironment with increased angiogenesis [15]. Recognizing and utilizing these molecular subtypes in GBM therapy could facilitate personalized treatment and improve patient outcomes. Research has shown that glioma cells exhibit a greater responsiveness to treatment through autophagy as opposed to apoptosis [16], making the autophagy pathway a compelling target in the GBM treatments. Also, according to a recent whole transcriptome expression data analysis, high expression levels of autophagy-related genes are detected in classical and mesenchymal subtypes, the more malignant tumor subtypes [17,18].

Autophagy is a natural cellular process that plays a crucial role in maintaining cellular homeostasis, adapting to stress conditions, and promoting cell survival [19,20]. There are three different primary types of autophagy, which include microautophagy, chaperone-mediated autophagy, and macroautophagy [21]. These pathways are differentiated by the method by which cytosolic cargoes are delivered to the lysosome [22]. Microautophagy involves the lysosome utilizing direct membrane protrusion to take up cytoplasmic material for degradation [23]. Microautophagy has gained significant attention in recent research endeavors. It not only relates to macroautophagy regulation [21] but also plays a role in cancer and neurodegenerative disorders [24,25,26,27]. Chaperone-mediated autophagy (CMA) is a selective form of autophagy that targets specific individual proteins for degradation [28,29]. CMA involves cytosolic proteins with KFERQ-like motifs that are recognized by chaperone HSC70, and these proteins are delivered to the lysosomal-associated membrane protein 2A (LAMP2A), which allows for the translocation of these proteins into the lysosome for degradation [30]. Quite a few proteins involved in neurodegenerative disorders, including α-synuclein [31,32,33] and β-amyloid precursor protein [32], contain this motif. Thus, inhibiting CMA in neurons impairs neuronal function and leads to the accumulation of harmful proteins, thereby raising susceptibility to Alzheimer’s disease [34]. Macroautophagy (autophagy) plays a key role in cell physiology and will be the focus of this review. Some of these roles include the degradation and recycling of damaged organelles, as well as the maintenance of normal cellular homeostasis [35]. The basic mechanism of autophagy has been well documented and involves the formation of a cytosolic double-membrane vesicle known as the autophagosome, which fuses with lysosomes to degrade its contents [36]. Macroautophagy generally occurs at low levels and can be further induced under specific stress conditions. Macroautophagy deteriorates cytoplasmic material into metabolites that can be used in biosynthetic processes or energy production for cell survival [37]. While autophagy primarily functions as a cytoprotective mechanism for degrading damaged organelles, uncontrolled autophagy can lead to detrimental consequences for the cell. 

Due to the involvement of autophagy in a diverse array of cellular functions, it must be tightly regulated. Autophagy can be upregulated in cells when they experience stressors and need to clear various toxic cytoplasmic materials or mobilize intracellular nutrients [38,39,40,41,42]. The inhibition or disruption of autophagy can lead to the accumulation of damaged organelles and harmful metabolic products within the cell [41,42,43,44]. Autophagy dysfunction is connected to a diverse list of human pathologies, including neuronal, lung, and liver dysfunction, heart disease, and cancer [37,45]. In particular, autophagy plays dual contrasting roles in cancer progression. It has been reported that autophagy can prevent tumor initiation by clearing toxic metabolites, which conserves the cellular genomic integrity and prevents cell proliferation [46,47]. However, established tumors can utilize autophagy to survive cellular stress [48,49,50], and this is believed to mediate therapy resistance.

Mutations in autophagy can lead to various pathological changes throughout the body [19,51]. Experimental evidence has linked autophagy to neurodegenerative diseases due to the presence of abnormal autophagosomes in the neurons of neurodegenerative diseases [52]. Autophagy impairment is reported to be involved in Parkinson’s disease by accumulating endogenous α-syn and LRRK2 proteins and causing progressive dopaminergic neuron loss [41]. Other evidence suggests that autophagy may play a neuroprotective role in neurodegenerative diseases by helping clear pathogenic protein aggregates [41,53]. In addition, autophagy has been associated with cancers in various organ systems [54,55,56,57]. By increasing resistance to the extreme stress developed within the tumor microenvironment, autophagy can aid cancer cells during chemotherapy [58,59,60]. For example, researchers have paired autophagy inhibition with autophagy modulation to induce cell death in cholangiocarcinoma [61]. This has been identified as a promising strategy for cholangiocarcinoma, and further understanding the role of autophagy in this strategy can pave the way for other aggressive cancer treatments.

Glioblastoma, the most common and aggressive brain tumor, is characterized by its highly invasive and therapy-resistant nature. Autophagy has been found to play a significant role in the various stages of glioblastoma development, including tumor initiation and progression, cell proliferation, and response to treatment. Elevated levels of autophagy have been associated with glioblastoma development and are correlated with poor glioblastoma survival [62]. Suppressing autophagy has shown promise in inhibiting glioblastoma development; for instance, the knockdown of key autophagy-related genes, such as Atg7, Atg13, or Ulk1, disrupts autophagy and inhibits glioblastoma development in KRAS-driven glioblastoma mouse models [63], indicating the critical role of autophagy in tumor initiation. In established tumors, conditions like hypoxia and nutrient deficiency can induce high autophagy activity. This autophagy activity enhancement could be induced by yes-associated protein (YAP)-mediated high mobility group box 1(HMGB1) translocation. When HMGB1 translocates from the nucleus to the cytoplasm, it enhances autophagy, thereby promoting GBM progression [63,64]. In contrast, autophagy overactivation implicates cell death and impedes GBM invasion. One of the mechanisms through which autophagy reduces glioblastoma invasion is by impacting N-cadherin. Studies have shown that membrane N-cadherin enhances cell focal adhesion and reduces cell migration [65], and autophagy increases N-cadherin membrane localization, thus suppressing glioblastoma invasion [66,67]. 

**Figure 1:**
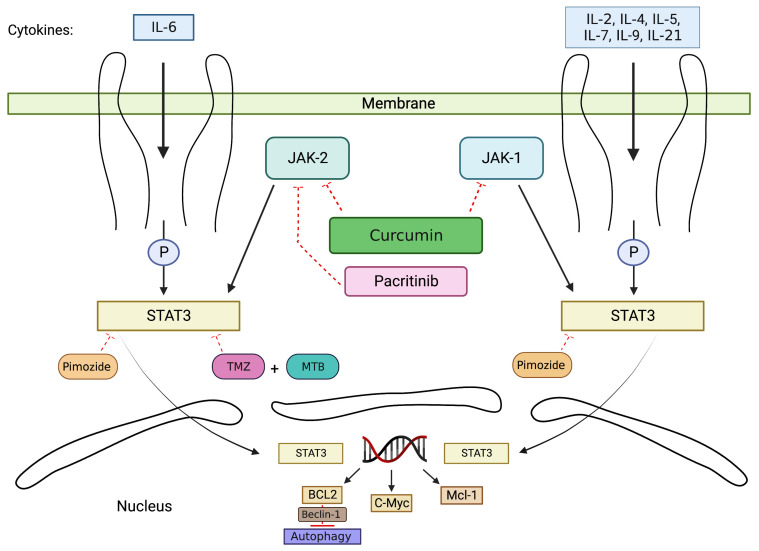
**Activating autophagy by targeting the JAK/STAT pathway.** The JAK/STAT pathway is activated when cytokines bind to cell membrane JAK receptors. The drugs discussed in this review are listed within the figure and include pimozide, temozolomide (TMZ), momelotinib (MTB), pacritinib, and curcumin. Pimozide, TMZ, and MTB function to inhibit STAT3, which leads to the downstream effect of autophagy induction. Curcumin inhibits both JAK-1 and JAK-2, which ultimately leads to autophagy induction. Pacritinib specifically inhibits JAK-2 and leads to autophagy induction as well.

**Figure 2:**
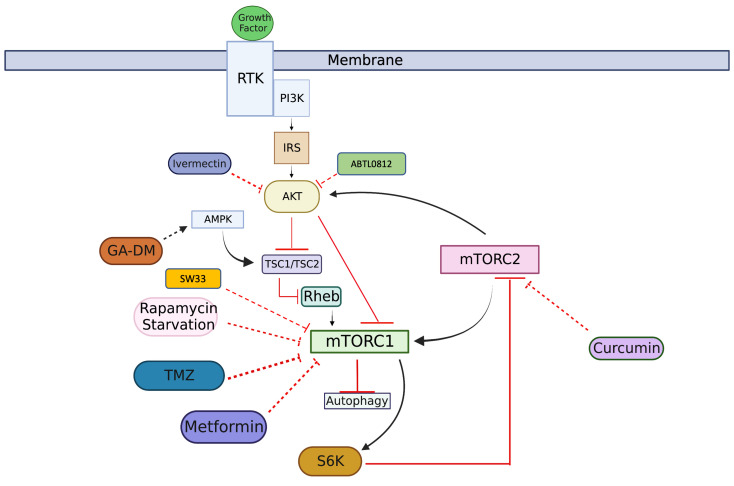
**Induction of autophagy by inhibiting the PI3K/AKT/mTOR pathway.** The PI3K/AKT/mTOR pathway is activated when growth factors activate Receptor Tyrosine Kinase (RTK), which then leads to activation of PI3K. The drugs affecting the PI3K/AKT/mTOR pathway are listed within the figure and include rapamycin, curcumin, ganoderic acid DM (GA-DM), ivermectin, ABTL0812, metformin, SW33, and temozolomide (TMZ). Rapamycin, TMZ, metformin, and SW33 inhibit mTORC1, which leads to autophagy induction. Curcumin inhibits mTORC2, which promotes induction of autophagy. GA-DM activates AMPK, which leads to activation of TSC1/TSC2 and, ultimately, autophagy induction. Ivermectin and ABTL0812 inhibit AKT, which leads to autophagy induction as well.

Therefore the role of autophagy in GBM development and progression is complex. On one side, autophagy induction can support GBM survival; however, when autophagy is upregulated beyond a certain threshold, it begins to inhibit cell proliferation and induce tumor cell death. Therefore, gaining a better understanding of the role of autophagy in GBM is crucial for the development of more effective and targeted treatment avenues. This review will provide an in-depth analysis of the current literature regarding autophagy-related pathways and mechanisms leading to glioma formation, along with current and future treatment modalities. The Janus kinase/signal transducer and activator of the transcription (JAK/STAT) signaling pathway regulates cell growth, survival, and differentiation, and the dysregulation of JAK/STAT signaling has been linked to cancer development. Similarly, the PI3K/Akt/mTOR pathway is also important in cell proliferation and tumor formation. Both of these pathways have been implicated in the regulation of autophagy [68,69,70]. In this review, we will focus on two pathways—the JAK2/STAT3 pathway (Figure 1) and the PI3K/AKT/mTOR pathway (Figure 2)—for the treatment of GBM. Given the presence of multiple GBM subtypes within the same patient and the ability of GBM tumor cells to switch between subtypes [71,72], the discussion will not distinguish treatments for specific subtypes. 

## 2. Exploring the Mechanism and Application of Autophagy Modulation through the JAK/STAT Pathway

### 2.1. JAK/STAT Signaling Pathway

JAK/STAT pathway is activated via Janus kinase/signal and heavily contributes to tumorigenic functions, including proliferation, anti-apoptosis, angiogenesis, stem cell maintenance, and immune suppression [73,74,75,76]. JAK-mediated pathway is activated when cytokines bind to JAK receptors located on the cell membrane, followed by receptor dimerization and cytoplasmic tail phosphorylation (Figure 1). The JAK family consists of non-receptor protein tyrosine kinases (PTKs), which include JAK1, JAK2, JAK3, and Tyk2 [70,77]. These kinases are upstream of STAT3 and are involved in the signal transduction of cytokines [70]. STAT proteins have SH2 domains that contain tyrosine phosphorylation sites, and their phosphorylation allows STAT to form dimers and alters STAT protein conformation to facilitate their DNA binding [78]. After tyrosine phosphorylation, STAT is translocated into the nucleus. In the nucleus, STAT proteins bind DNA elements and regulate the transcription of associated genes, such as Bcl2, c-Myc, and Mcl-1 [78,79,80]. The STAT family is composed of seven members, which include STAT1, STAT2, STAT3, STAT4, STAT5A, STAT5B, and STAT6 [81]. STATs 1, 2, 4, and 6 play limited roles, while STAT3 and STAT5 are involved in more functions, such as resistance to treatments [82,83]. STAT3 serves as an intracellular signaling hub and can be activated when JAK2 is stimulated by various cytokines (IL-6) and growth factors. Once JAK2 is activated, it can phosphorylate STAT3 at the tyrosine 705 residue [73,84]. STAT3 is the primary transcriptional regulator of autophagy genes in the nucleus [70,85], and it can inhibit autophagy via the upregulation of BCL2 [85]. Experimental evidence has shown that the inhibition of STAT3 leads to autophagy upregulation [85]. STAT3 has been observed in glioblastoma multiforme (GBM), and STAT3 activation is correlated with a poor prognosis in GBM [86]. 

### 2.2. Drugs Induce Cell Apoptosis via Inhibition of JAK/STAT Pathway

Along with understanding the molecular mechanism of the JAK/STAT pathway, it is also important to understand the drugs that can influence this pathway. Therefore, we can modulate autophagy during cancer treatment. Pimozide is an antipsychotic drug that functions as a dopamine antagonist, but it has also been shown to promote tumor apoptosis [78,87,88]. Through studies on GBM cells, such as U87, U251, Daoy, and GBM 28, pimozide has been shown to promote autophagy-mediated apoptosis in vivo and in vitro by inhibiting the JAK/STAT3 pathway [78]. When the JAK/STAT3 pathway is inhibited, the development of malignancies such as brain tumors is suppressed. This is because STAT3 regulates the transcription of various oncogenes, such as Mcl-1, c-Myc, and Bcl-2 [89]. Bcl-2 is cited as one of the anti-apoptotic proteins and forms a complex with Beclin 1 [90,91]. When Bcl-2 complexes with Beclin 1, Beclin 1 is inactivated, causing the inhibition of autophagy-mediated apoptosis via the Bcl-2-Beclin1 complex [92,93,94]. When pimozide inhibits the JAK-STAT3 pathway, autophagy is released from its inhibition and autophagy-mediated apoptosis is promoted [78]. Overall, pimozide shows promise as an agent that can inhibit glioma growth through STAT3 inhibition. However, additional studies are needed to determine the side effect profile of prolonged pimozide administration.

Another potential enhancer of glioma treatment that has been researched is curcumin. Curcumin is an herbal supplement extracted from turmeric with lipophilic characteristics, which allows it to penetrate the blood–brain barrier and potentially affect several glioma tumor processes, such as proliferation, cell death, metastasis, and chemoresistance [95,96,97,98,99]. One of the signaling pathways that curcumin affects is the JAK1,2/STAT3 pathway [99,100,101]. Curcumin can downregulate the JAK1,2/STAT3 signaling pathway and its downstream targets. Klinger’s group carried out an in vitro study involving murine glioma cell lines (Tu-2449, Tu-9648, and Tu-251) to elucidate how curcumin disrupts molecular signaling. When the murine glioma cell lines were treated with curcumin, it led to the dephosphorylation of JAK1 and JAK2 in a dose-dependent manner. This change inactivated downstream STAT3 and inhibited the transcription of STAT3 target genes [102,103]. Another portion of this study utilized C6B3F1 mice as an in vivo mouse model and provided these mice with a high-fat diet supplemented with or without curcumin over a 7-day period. The mice were also implanted with either Tu-2449 or Tu-9648 glioma cells and continued their respective diets across the 7-day period. The results of this sub-study showed that 15% of Tu-2449-implanted mice who received curcumin-fortified diets had tumor-free long-term survival compared to 0% of non-curcumin-diet-supplemented animals. With regard to the Tu-9648-implanted mice who were fed curcumin-enriched diets, they experienced a 38% higher long-term tumor-free survival compared to the control animals [102]. These findings imply that curcumin’s potential benefits could fluctuate depending on the specific glioma cell type. Unfortunately, there have been limited studies using human participants to determine the safety and efficacy of curcumin within the context of gliomas. Additional clinical data on curcumin’s treatment of GBM would be valuable for assessing its therapeutic potential in GBM treatment. 

### 2.3. Drugs Enhance the Efficacy of TMZ via JAK/STAT Pathway

Temozolomide (TMZ) is considered the first-line drug for the clinical treatment of gliomas [94,104]. TMZ can induce autophagy by initiating DNA damage and triggering the activation of the ATM/AMPK/ULK1 signaling pathway, which, in turn, promotes autophagy [105,106]. However, TMZ resistance is one of the most difficult challenges to overcome in GBM treatment [107,108]. In order to enhance the chemosensitivity of GBM to TMZ, momelotinib (MTB) is used in combination with TMZ [108,109,110]. MTB is an aminopyrimidine derivative and has been shown to inhibit the phosphorylation of JAK2 and STAT3 in GBM U251 cells [111]. During the treatment, MTB enhances the chemosensitivity of GBM to TMZ via the inactivation of the MTB/JAK2/STAT3/Bcl2 axis [109,110]. This increased chemosensitivity of GBM cells was demonstrated via studies on mouse xenograft models. In the study, co-treatment with MTB and TMZ on GBM U251 cells led to enhanced autophagy followed by apoptosis due to the inhibition of JAK2 and STAT3 phosphorylation [111]. This particular experiment showed that MTB and TMZ co-treated xenografts significantly decreased tumor weight compared with TMZ treatment alone. This suggests that MTB could suppress cell growth and overcome chemoresistance through the inactivation of the JAK2/STAT3 pathway, thereby leading to an increase in autophagy and triggering autophagy-mediated cell apoptosis. 

Pacritinib is a JAK2 inhibitor with blood–brain barrier permeability and shows promising effects as an inhibitor that can be paired with TMZ to improve the efficacy of TMZ [104,112]. The synergistic effect of combination treatment with pacritinib and TMZ was demonstrated in patient-derived GBM brain-tumor-initiating cells (BTICs) [112]. When BTICs were treated with either 10 μg/mL of TMZ, 1 μM of pacritinib, or a combination of 1 μM pacritinib and 10 μg/mL of TMZ, Western blot protein analysis detected a significant increase in STAT3 activation in the TMZ-only treatment group. This suggests that utilizing TMZ treatment alone can increase STAT3 activation, which can promote autophagy inhibition and play a role in TMZ resistance. However, with the combined treatment of TMZ and pacritinib, this compensatory increase in STAT3 signaling was negated [112]. Moreover, Jensen’s group also tested the impact of pacritinib on other signaling pathways and found that at lower concentrations, such as 1 μM, pacritinib could significantly decrease STAT3 activation and did not impact other signaling pathways. However, when pacritinib was used at higher concentrations, such as 5 and 10 μM, it also decreased p-Akt S473 and p-p44/42 MAPK signals [112]. This experiment highlights the specificity of pacritinib in reducing JAK2-mediated STAT3 activity [112,113] and suggests that higher concentrations of pacritinib have additional off-target signaling impact in addition to the JAK2/STAT3 pathway. Although clinical phase trial data are necessary, the preclinical experimental data suggest that pacritinib, when used in combination with TMZ, could potentially extend the survival of GBM patients. 

## 3. Unveiling Autophagy Modulation Mechanism and Application via PI3K/AKT/mTOR Pathway

### 3.1. PI3K/AKT/mTOR Signaling Pathway

In addition to the JAK/STAT pathway, the phosphatidylinositol 3-kinase /AKT/mammalian target of rapamycin (PI3K/AKT/mTOR) pathway is also involved in autophagy modulation [36,114,115,116]. The PI3K/AKT/mTOR pathway is a modulated signaling pathway in many cancer types, known to regulate different cellular processes such as cell survival, proliferation, growth, metabolism, angiogenesis, and metastasis [117,118,119]. mTOR is a serine/threonine protein kinase, and it is a downstream effector of PI3K and AKT signaling (Figure 2). There are two mTOR complexes: mTORC1 and mTORC2. The function of mTORC2 is not well understood, but it is responsive to growth factors for controlling cytoskeleton organization [120,121]. Activated mTORC2 plays a modulatory role in relation to mTORC1 and phosphorylates AKT at Ser473. The phosphorylated AKT can inhibit TSC1/2, resulting in the hyperactivation of mTORC1 through Rheb (Figure 2). mTORC1 is considered a major negative regulator of autophagy, and the PI3K/AKT pathway is a major upstream modulator of mTORC1 [122]. The suppression of the PI3K/AKT/mTOR pathway promotes autophagy, while the activation of this pathway inhibits autophagy [36]. The PI3K/AKT/mTOR pathway regulates synaptic plasticity in the brain and plays an important role in the development of brain structure; thus, the dysregulation of this pathway is commonly reported in neurodegenerative pathologies [36]. As previously stated, multiple genes of the PI3K/AKT/mTOR signaling pathway are commonly altered in human cancers, such as glioblastomas, making it a clinically relevant molecular therapeutic target [123,124]. 

### 3.2. Drugs Induce Autophagy-Mediated Cell Death by Inhibiting AKT/mTOR Pathway

Several drugs are involved in the PI3K/AKT/mTOR signaling pathway, including rapamycin, an mTORC1 inhibitor and autophagy modulator [125]. Rapamycin is produced by *Streptomyces hygroscopicus*, and was initially used as an antifungal compound [126,127]. Several studies were carried out to determine whether rapamycin could induce autophagy. In one of the studies, U87-MG cells were treated for 3 days with rapamycin at a dosage of 100 nmol/L, and the ultrastructure of the cells was visualized through electron microscopy [128]. EM revealed that the cells treated with rapamycin developed numerous autophagic vacuoles, but not chromatin fragmentation [128]. These results indicate that rapamycin exerts its effects through autophagy induction as opposed to apoptosis. Rapamycin functions by inducing autophagy through the inhibition of mTORC1 [125]. This autophagy induction initially leads to an antitumor effect, which helps sensitize radioresistant cancer cells to radiotherapy [126]. Due to the heterogeneous nature of cancer cells, only a subset of cancer cells can achieve senescence when treated with a combination of rapamycin and radiation [129]. This augmentation of autophagy in susceptible cells led to tumor suppression. Overall, clinical trial outcomes using rapamycin have not been positive for most types of cancers, including glioblastomas [126]. A potential mechanism hindering rapamycin cancer treatment could be the loss of the negative feedback circuit from PI3K/AKT/mTORC1 signaling to the PI3K/AKT/mTORC2 signaling axis, which is mediated via ribosomal protein S6K [130] (Figure 2). S6K is a member of the serine–threonine AGC kinase family and can inhibit mTORC2 [131]. When mTORC1 is inhibited, S6K is subsequently inhibited, and this allows for the PI3K/AKT/mTORC2 signaling axis to remain active in cancer cells [126]. Since rapamycin and its analogs, such as everolimus, have limited activity against mTORC2, there is incomplete downstream inhibition of mTORC1 targets [132]. This incomplete blockade of mTORC2 may lead to the inhibition of autophagy. 

Along with the drugs previously mentioned, ivermectin (IVM), an antiparasitic drug, has been considered a promising anticancer drug [133]. Ivermectin therapy has been shown to enhance autophagy in glioma cells by increasing the production of autophagosomes and activating autophagy-related genes and proteins [134,135,136]. Liu’s group reported the effect of ivermectin on cell growth and metabolism, as well as autophagy modulation [136]. Their group utilized U251 and C6 GBM cells to determine the impact that IVM had on autophagy. Western blot showed p-AKT (Ser473)/AKT and p-mTOR (S2448)/mTOR presented a dose-dependent reduction with the administration of IVM at 0, 5, 10, and 15 μM [136]. According to these findings, IVM can inhibit the AKT/mTOR pathway, resulting in autophagy induction and decreased cell growth. The findings indicate ivermectin could induce autophagy-mediated cell death in glioma cells by inactivating AKT/mTOR signaling [136].

ABTL0812 is an oral anticancer drug currently in phase II clinical trials for the treatment of glioblastoma. ABTL0812 application increases TRIB3 expression in GBM cells, which, in turn, inhibits the AKT/mTORC1 pathway, leading to cell death through autophagy. In in vivo studies using intra-brain xenograft tumor models, a combination of ABTL0812 with radiotherapy and temozolomide (triple combo) has shown a synergistic effect, resulting in increased disease-free survival in mice [15]. 

A similar mechanism has been observed in the treatment of GBM with a Sinomenine ester derivative, sino-wcj-33 (SW33). Sinomenine is an alkaloid extracted from the Chinese herb *Sinomenium acutum*, and shows the potential for cancer treatment. When U251 cells were treated with SW33 for 24 h, transmission electron microscopy revealed the presence of autophagosomes and autolysosomes inside the tumor cells [137]. Western blotting analysis showed a reduction in the ratio of p-mTOR to mTOR, indicating that cell death is a result of increased autophagic response mediated by the inhibition of the AKT/mTOR pathway. 

### 3.3. Drugs Decrease Cell Proliferation by Targeting AKT/mTOR Pathway

Autophagy inhibition in cancer cells can promote tumor development because genomic defects can accumulate within the cell and foster an environment for cancer cell growth [138]. As mentioned before, curcumin is a promising nutraceutical compound that provides effective treatment for GBM by downregulating the JAK/STAT3 pathway [95,102]. Surprisingly, curcumin is reported to target the AKT/mTOR pathway as well. An in vitro study with U87 human glioma cells treated with 5–10 μM curcumin showed that the cells remain arrested in the G2/M stages, subsequently inhibiting cell proliferation [102]. Additional studies on glioblastoma cell line U251 showed that when cells were treated with curcumin, phosphorylation levels of AKT and mTOR were significantly decreased [139]. This suggests that curcumin led to autophagy induction, and can lead to autophagy-mediated cell death in GBM cells by inhibiting the AKT/mTOR pathway [99]. Although current studies have shown that curcumin exhibits promising anticancer mechanisms, its efficacy is hindered by its low bioavailability, poor absorption, and rapid systemic elimination [99,140]. Future clinical trials with larger sample sizes could focus on optimizing nano formulations of curcumin to improve drug bioavailability and understanding its synergies with standard chemotherapeutic drugs [99].

Ganoderic acid DM (GA-DM) is a lanostanetriterpene that is isolated from the medicinal mushroom Ganoderma lucidum [141]. It shows potential antitumor activity in different cancers, including human glioblastoma [142]. Ganoderic acid DM induces G1 phase cell cycle arrest and activates p53 in the intrinsic apoptotic pathway [143]. Studies have shown that autophagy could be induced through GA-DM via the activation of the AMPK pathway followed by mTORC1 inhibition and the subsequent induction of autophagy [144,145]. This indicates that GA-DM could induce autophagy through modulating mTORC1. In addition, laboratory trials have confirmed GA-DM upregulates Beclin-1 and LC3. LC3 is an abbreviation for microtubule-associated proteins 1A/1B light chain 3 (MAP1LC3 or simply LC3), and it plays a role in the autophagy process [146]. By increasing the levels of Beclin-1 and LC3, GA-DM enhances their participation in autophagy induction, thereby promoting the autophagy process [147,148]. As a result, GA-DM effectively induces autophagy by inhibiting the PI3K/AKT/mTOR pathway [145].

Adding to the list of drugs targeting the AKT/mTOR pathway, metformin has been shown to be an anti-glioma agent [149,150,151]. The proliferation of human glioma cells is reported in relation to the effects of metformin [152]. For example, metformin decreases cell proliferation, and the effect could be partially reversed via the daily replacement of old media with new media [152]. It is speculated that off-target effects such as increased glycolytic pathway activity, which can cause media acidification and toxicity, or higher glucose consumption, which can cause glucose depletion and cell starvation, may be the cause of metformin-induced toxicity [153,154]. It was also discovered that PTEN (Phosphatase and Tensin Homolog deleted on Chromosome 10) wild-type cells (LN18 and SF767) were more sensitive to metformin than PTEN mutant cells (U87 and U251), which suggests that metformin can suppress AKT more effectively in PTEN WT cells, resulting in autophagy activation followed by cell apoptosis [152]. Additionally, treatment with metformin decreases the quantity of GBM cells undergoing cell division and enhances cell cycle arrest, which inhibits cell proliferation. Time course studies have indicated that metformin first causes cell cycle arrest, then autophagy induction, and, finally, cell death [136,152]. Sesen’s group demonstrated, through flow cytometry, that metformin-treated glioma cells showed greater acridine orange staining than control cells. Since acridine orange is a marker of vacuole acidification [155], these results indicated that metformin induces autophagy through the development of autophagosomes [152]. According to this experiment, vacuole acidification primarily occurred 24 h after metformin therapy, and this metformin-induced vesicle acidification could be abolished via the late-phase autophagy inhibitor bafilomycin A1, demonstrating the occurrence of autophagic flux [152]. Furthermore, the use of metformin also increased the expression of autophagy-related proteins in the glioma cells, including LC3b-II and Beclin-1. A drop in p62 levels, a sign of activated autophagy [156], was also seen in some cell lines. These results imply that metformin administration causes human glioma cells to undergo autophagy through the inhibition of the mTOR pathway [152]. A similar anti-proliferative effect has been shown on lymphoma cells, where cell growth can be inhibited through the blockage of the mTOR pathway [157]. According to recent studies, metformin decreases mTOR activity and mitochondrial respiration through enhancing the PRAS40-RAPTOR connection, which is independent of AMPK [158]. Therefore, it is implied that metformin induces cell death by inhibiting the mTOR pathway. 

Our group also tried different strategies in the treatment of glioblastoma by inducing autophagy. Niclosamide is an FDA-approved drug used to treat tapeworm infections. Recently, it also showed promising chemotherapeutic effects. In our study, when niclosamide was applied to U87 MG cells at concentrations of 0 to 40 μM for 24 h, cell proliferation/viability showed a significant reduction in a dose-dependent manner. Monodansylcadaverine (MDC) is a specific autophagolysosome marker and is used to analyze the autophagic process. In a further study, we confirmed niclosamide could induce autophagy via monodansylcadaverine (MDC) staining. This autophagy induction could be mediated through both the JAK2/STAT3 pathway and the PI3K/AKT/mTOR pathway, as niclosamide demonstrated down-regulation in the expression of both PI3K/AKT and STAT3 signal transduction pathways [159]. Additionally, we also tried the application of Cissus quadrangularis for U87 MG cells. *Cissus quadrangularis* is commonly known as Hadjod, a vining plant native to South Asia and Africa. *Cissus quadrangularis* has been used for the treatment of osteoporosis and osteoarthritis. Recently, *Cissus quadrangularis* demonstrated significant antioxidant and anticancer effects. Our data showed that treatment with *Cissus quadrangularis* induced cytotoxicity in U87 MG cells and triggered autophagy induction, as indicated by increasing LC3 II expression. This autophagy induction could be induced by suppressing the JAK2/STAT3 pathway, as STAT3 expression and phosphorylation were inhibited via *Cissus quadrangularis* treatment in a dose-dependent manner [160]. 

## 4. Future Perspectives for Autophagy Studies

Through extensive research regarding autophagy and its underlying mechanism, we have gained a better understanding of its multitude of functions in maintaining cellular homeostasis and disease pathogenesis. Autophagy is a highly conserved cellular process that allows cells to adapt to a variety of conditions. Autophagy is a dynamic process and multiple networks can modulate and regulate this process. The pathways we discussed were the JAK/STAT and PI3K/AKT/mTOR pathways. Although there are additional pathways that serve to modulate autophagy activity, we focused on these pathways to highlight the role that autophagy plays in brain tumors. Autophagy has been known to play dual contrasting roles in cancer. In the initial phases of tumor formation, autophagy may have a tumor-suppressive role. Since autophagy removes damaged organelles and misfolded proteins within the cell, this minimizes the risk of genomic instability and potential tumor development. However, within established tumor cells, autophagy induction can be utilized as a survival mechanism. This allows for the established tumor cells to adapt to nutrient-stressed conditions and promotes resistance to therapeutics. However, when autophagy is overactivated and passes the specific threshold, autophagy-mediated cell death begins. Navigating the paradoxical role that autophagy plays in cancer cells has been one of the biggest challenges in developing effective therapies. 

We centered our discussion around the role of autophagy modulation in current therapeutic interventions and strategies for glioma treatment. This review examined the challenges involved in current therapeutic interventions and highlighted the potential synergies that different treatments may have when used in combination. A specific area that warrants continued research is understanding the molecular mechanisms of the synergies that combination autophagy modulation treatments may have in improving the prognosis of gliomas. Additionally, clinical trials with large sample sizes should be conducted to understand the tangible clinical benefit that autophagy modulation treatments may have on gliomas. As research continues to progress in autophagy modulation, we can gain a better understanding of the pathways underlying its therapeutic effect and utilize this knowledge to develop targeted novel clinical applications for glioma treatment.
